# The characteristics, experiences and perceptions of naturopathic and herbal medicine practitioners: results from a national survey in New Zealand

**DOI:** 10.1186/s12906-015-0616-5

**Published:** 2015-04-10

**Authors:** Phillip Cottingham, Jon Adams, Ram Vempati, Jill Dunn, David Sibbritt

**Affiliations:** Wellpark College of Natural Therapies, PO Box 78–229, Grey Lynn, Auckland 1245 New Zealand; University of Technology Sydney, Sydney, Australia

**Keywords:** Naturopathy, Herbal medicine, National survey, Perceptions, Practice characteristics, Integrative practice, Referrals

## Abstract

**Background:**

Despite the popularity of naturopathic and herbal medicine in New Zealand there remains limited data on New Zealand-based naturopathic and herbal medicine practice.

In response, this paper reports findings from the first national survey examining the characteristics, perceptions and experiences of New Zealand-based naturopaths and herbal medicine practitioners across multiple domains relating to their role and practice.

**Methods:**

An online survey (covering 6 domains: demographics; practice characteristics; research; integrative practice; regulation and funding; contribution to national health objectives) was administered to naturopaths and herbal medicine practitioners. From a total of 338 naturopaths and herbal medicine practitioners, 107 responded providing a response rate of 32%. Data were statistically analysed using STATA.

**Results:**

A majority of the naturopaths and herbal medicine practitioners surveyed were female (91%), and aged between 45 and 54 years. Most practiced part-time (64%), with practitioner caseloads averaging 8 new clients and over 20 follow-up clients per month.

Our analysis shows that researched information impacts upon and is useful for naturopaths and herbal medicine practitioners to validate their practices.

However, the sources of researched information utilised by New Zealand naturopaths and herbal medicine practitioners remain variable, with many sources beyond publications in peer-reviewed journals being utilised. Most naturopathic and herbal medicine practitioners (82%) supported registration, with statutory registration being favoured (75%). Integration with conventional care was considered desirable by the majority of naturopaths and herbal medicine practitioners surveyed (83%).

Naturopaths and herbal medicine practitioners feel that they contribute to several key national health objectives, including: improved nutrition (93%); increased physical activity (85%); reducing incidence and impact of CVD (79%); reducing incidence and impact of cancer (68%).

**Conclusions:**

There is a need for greater understanding and communication between practitioners of conventional care and naturopathic and herbal medicine which could support informed, coordinated and effective health provision within the New Zealand health care system. There is a need for further in-depth research examining naturopaths and herbal medicine practitioners’ perceptions and practices, to provide insights of benefit to all those practising and managing health services as well as those directing health policy in New Zealand.

## Background: naturopathy and herbal medicine in New Zealand

There has been a worldwide increase in complementary and alternative medicine (CAM) use [1-3]. The most recent data from New Zealand (NZ) reports that in the twelve months prior to October 2006 at least 1 in 5 adults visited a CAM practitioner [4]. Naturopathic and herbal medicine practitioners (N/HMPs) constitute a substantial component of CAM health care provision in NZ [5]. The Natural Health Council [6] and Natural Health Practitioners [7] hold voluntary registers in naturopathy/herbal medicine (N/HM) and data from the 2006 Census [8] shows that naturopathic medicine makes up 0.6% of the NZ healthcare workforce (no separate census data is available for HMPs, who are combined with other complementary practitioners). These census results show that the total NZ CAM workforce is 2,055 [8], with naturopathic medicine constituting 21% of this workforce.

N/HM exist predominantly outside publically funded health care and beyond the practices of the conventional medical profession in NZ [9]. CAM more generally, of which N/HMPs form a substantial component, currently attracts no Government regulation in NZ, despite advice from a ministerial committee [10]. A 2006 survey of NZ GPs showed that the rate of referral from GPs to naturopaths was 12.3% and the rate of referral from GPs to HMPs was 9.7%. However, this same 2006 study also identified the GPs opinions about the benefit of N/HM was 33% and 27.7% respectively [11].

### Practices and perceptions of naturopathic and herbal medicine practitioners in Australasia

Some research, undertaken outside NZ, has examined the practices and perceptions of N/HMPs. An Australian survey of N/HMPs found that while 76% of practitioners had undergraduate or post-graduate qualifications, only 29% sourced information from peer reviewed scientific journals [12]. Another study of N/HMPs in Australia found that most of their patients (74%) were self-referred (word of mouth) and that most N/HMPs surveyed practiced part-time with mean clinical hours of 23.8 per week [13].

In North America NM practice patterns have been studied, finding that NMPs saw, on average, 31.6 patients per week; with 70% ordering diagnostic and screening tests [14]. Another study investigated NMs practitioner’s relationship with conventional care, with 22% in frequent communication with primary care physicians and 30% reporting lack of cooperation, leading to lack of access to medical records. This study also reports on NMs experience with scientific research, with 30% indicating they have coordinated or conducted research [15].

Yet, to date, the characteristics, perceptions and experiences of N/HMPs in NZ remain beyond empirical investigation. Such research can offer important information necessary to help those providing and managing health practices and developing health care policy, to ensure safe, effective and coordinated patient care for all New Zealanders. In response to this significant research gap, this paper reports findings from the first cross-sectional survey examining the characteristics, experiences and perceptions of N/HMPs in NZ. These descriptive data reveal information about the additions N/HMPs make to the healthcare workforce. Such research could provide background information for further studies that could effectively respond to the call from the NZ Ministerial Advisory Committee of Complementary and Alternative Health that there is a need for “quality, unbiased information on the safety and efficacy of CAM modalities, and for further work to be done to identify and address consumers’ needs for information about the practice of CAM in NZ” [10].

## Methods

A survey was administered to a range of CAM practitioners (including N/HMPs, homeopaths, massage therapists plus others) in NZ to examine their characteristics, perceptions and experiences relating to a range of key issues. This paper reports the analysis relating to the responses of the N/HMPs in the survey.

The study utilised an on-line survey via Survey Monkey. The survey was structured around seven domains: demographic practitioner data; practice and referral characteristics; academic qualifications; perceptions and experiences regarding research, integration and registration and alignment to the NZ health strategy [16]. Ethics approval was granted by the Ministry of Health (MoH) National Ethics Advisory Northern A committee and the Human Research Ethics Committee, University of Technology Sydney, Australia. Informed consent was implied by participation as per the NZ National Ethics Committee Guidelines [17].

### National sample of CAM practitioners

Participants were recruited through the two main registering bodies for CAM practitioners in New Zealand: the Natural Health Council and the Charter of Health Practitioners (now Natural Health Practitioners NZ). This recruitment method is similar to that employed in other surveys of naturopathic and herbal medicine practitioners [12,14,18]. Both registering bodies agreed to administer the survey via their affiliated associations, (*n* = 338 for N/HMP associations). Upon receiving the association membership figures, each association was allocated specific codes (to avoid duplicate responses). These codes were subsequently allocated to individual members by their own associations, along with the web address of the survey, ensuring anonymity of the respondents. As not all NZ naturopaths identified in the census [8] are registered with an association, the percentages used in the analysis were only calculated for practitioners who received an invitation to complete the survey. This does not represent the sum total of NZ naturopaths.

### Survey measures

The survey examined demographic data, specifically age, gender, ethnicity and practice location identified by NZ District Health Board (DHB), and whether the N/HMPs’ practice was in a city, urban or rural location(s). Practitioner characteristics examined in the study were: average caseloads, years of professional experience, highest level of qualification and attitudes to research. The survey also asked about the N/HMPs’ attitudes to integration and N/HMPs’ modes and rates of referral to a range of conventional health care practices. N/HMPs were asked whether they considered that they should be registered and whether that registration should be statutory or voluntary. This section also included questions on whether N/HMPs should receive Government or private funding (or a combination of both) as well as their own use of indemnity insurance schemes. The survey also included a question examining N/HMPs perceptions of their alignment to the public health goals stated in NZ Health Strategy [16].

### Analysis

Initial descriptive analysis was undertaken for all responses using means and standard deviations or frequencies and percentages where appropriate. A chi-square test was used to examine the association between two categorical variables and a Student’s t-test was used to test for differences between means across a binary variable. A p-value of <0.05 was set to indicate statistical significance. All analyses were undertaken using STATA software. Data for referrals were analysed using 5 or less as the first category, and then 6 or more for the second for pragmatic reasons.

## Results

A total of 107 N/HMPs responded to the survey representing a response rate of 32% for these modalities. Most participants were female (91%) and a significant portion of participants were aged 45 to 54 years (38%), with only 12% aged 35 years or younger. Most practitioners (53%) had an undergraduate or postgraduate degree (in a related discipline), with their base N/HM qualification predominantly at a Diploma or Advanced Diploma level (82%)^a^. Modalities (other than naturopathy and herbal medicine) practiced included: nutritional supplementation (71%); nutritional counselling (63%); relaxation massage (35%); therapeutic massage (29%); homeopathy (19%); aromatherapy (14%) and spiritual healing (9%).

### Practice characteristics

Clinical practice and practice management constituted the majority of professional hours spent per week (mean = 15 hours) by the N/HMPs, followed by: manufacturing and retail-related tasks (mean = 9 hours); education-related tasks (mean = 3 hours); administration tasks (mean = 3 hours); research (mean = 1 hour); training (mean = 1 hour); and other tasks (mean = 1 hour).

Most N/HMPs self-reported that they were located in urban areas (84%), with practices mainly in solo clinics (56), but also based in multi-disciplinary clinics alongside a range of CAM and non-CAM practitioners (33%) and other types of practices, including health food stores and pharmacies (11%). The majority of the respondents (60%) had more than 10 years of experience in practice. Only 26% of the N/HMPs practiced full-time, with 64% practicing part-time and 10% occasionally. Most practitioners had utilised more than one therapy in their practice (83%), with only 17% specialising in a single therapy or area of practice. The average new-patient caseload (per month) was 8.0 clients, while the average follow-up caseload was 20.3 clients (per month). Consultation length for new patients was reported as 76.2 minutes on average, whereas most follow-up consultations were an average of 37.1 minutes.

### Research

Most N/HMPs (80%) perceived research^b^ as being useful for validating their practice, with ninety-three per cent placing high value on research, and seventy-eight per cent identifying it as of high or moderate impact on their practice behaviour.

Whilst most N/HMPs (95%) utilised client-reported changes of symptoms as a main measurement of outcome, 60% of respondents employed blood tests and medical parameters, and 38% used other quantifiable measures, such as Health Related Quality of Life, as indicators of therapeutic effectiveness and 33% N/HM specific assessment methods (such as iridology or live blood analysis). Whilst 60% of practitioners agreed they had the skills to identify and analyse published research, and make meaningful clinical decisions based on this source of evidence, 26% identified themselves as having the skills to actually conduct research. Of those N/HMPs who reported that they had no skills in interpreting research, time (38%) and financial restraints (26%) were the main reasons given for this situation. However, only 30% of respondents reported that they remained up-to-date with research findings and only 35% reported using peer-reviewed scientific journals to keep up to date with knowledge in their field. Respondents were asked if they kept up-to-date with research findings and, if they did, where did they access information about research. Of those participants who did access research material, the sources of researched information were as follows: 82% from attending manufacturer’s/supplier’s seminars; 72% via newsletters from manufacturers and suppliers; 67% via Google search engine web articles; 51% from news articles; 50% via online scientific journals; 49% from health-specific magazines; 43% via association meetings; 35% via peer-reviewed scholarly journals; 20% from attending scientific presentations; 19% from attending international conferences; and 18% via reading trade journals.

No correlation was found between age, years of experience, CAM related activities, case-loads or educational qualification in relation to either using researched information to validate practices or in relation to the impact of research on practice (see Table 1).

**Table 1:** **The association between naturopathic and herbal medicine practitioner characteristics and aspects of research**

**Practitioner and practice-related characteristics**		**Using research for validating practice**			**Research impact on practice**		
		**Useful** **(n = 93)**	**Not useful** **(n = 2)**	**p-value**	**High/Moderate** **(n = 84)**	**Low ** **(n = 17)**	**p-value**
		**Mean (SD)**	**Mean (SD)**				
**Years of experience**		11.1 (9.0)	3.5 (3.5)	0.240	2.7 (1.7)	2.9 (1.3)	0.643
**Hours spent on CAM-related training**		1.5 (1.1)	1.9 (1.6)	0.403	1.4 (1.1)	2.0 (1.8)	0.205
**Hours spent on administrative tasks**		2.1 (1.6)	1.5 (1.1)	0.181	2.0 (1.5)	2.0 (1.5)	0.644
**Average case-Load per month (all)**		31.4 (29.6)	32.2 (29.8)	0.908	31.5 (30.5)	26.0 (23.0)	0.488
		**%**	**%**		**%**	**%**	
**Age**	**22-44**	36.4	100	0.066	38.1	37.5	0.964
	**>44**	63.6	0.0		61.9	62.5	
**Education diploma**		47.3	33.3	0.627	42.9	46.7	0.791
Under-graduate		30.9	40.0		35.7	26.7	
Post-graduate		21.8	26.7		21.4	26.6	
**Sufficient skills to conduct research**	Yes	37.9	50.0	0. 728	37.5	41.7	0.788
	No	62.1	50.0		62.5	58.3	
**Sufficient skills to Yes**		58.0	100	0.396	60.7	52.9	0.552
**Interpret research No**		42.0	0.0		38.3	47.1	

Similarly, no correlation was found between skills to either interpret or conduct research and the validation or impact of research upon the N/HMPs’ practice (Table 1).

Most N/HMPs (70%) identified a need for a national CAM research unit in NZ, which was seen as a useful development to help provide guidance regarding: funding sources and opportunities (58%); developing study protocols and designs (54%); research methodology (51%); networking (50%); and collaboration (48%).

### Regulation and funding

Registration was supported by a significant majority (82%) of N/HMPs. Of the 88 practitioners who favoured some form of registration, 75% were in favour of statutory registration^c^ and 25% voluntary registration. No relationship was found between N/HMPs’ attitudes to registration and practitioner age (*p* = 0.735), years of experience in practice (*p* = 0.239), or academic qualification (*p =* 0.348) (Table 2).

**Table 2:** **The association between naturopathic and herbal medicine practitioner characteristics and aspects of regulation and funding**

**Practitioner characteristics**		**In favour of registration**			**In favour of combined government and private funding**		
		**Yes (n = 88)**	**No (n = 19)**	**p-value**	**Yes (n = 74)**	**No (n = 33)**	**p-value**
		**Mean (SD)**	**Mean (SD)**				
**Years of experience**		2.8 (1.7)	2.8 (1.7)	0.239	2.8 (1.6)	2.5 (1.8)	0.353
		**%**	**%**		**%**	**%**	
**Age**	**22-44**	37.9	42.1	0.735	37.8	40.6	0.787
	**>44**	62.1	57.9		62.2	59.4	
**Education**							
Diploma		41.9	25.0	0.348	42.9	50.0	0.813
Under-graduate		38.7	25.0		35.7	27.8	
Post-graduate		19.4	50.0		21.4	22.2	

Most N/HMPs favoured some practice subsidy funding being available, with 69% in favour of a combination of Government and private (insurance) subsidies. Sixty-one per cent of the naturopaths and herbal medicine practitioners had professional indemnity insurance. There was no significant association between funding preference and age (*p* = 0.787), practice years (*p* = 0.353) or academic qualifications (*p* = 0.813) (Table 2).

### Integrative practice

Most N/HMPs (83%) favoured integration with conventional care providers. Specifically, the percentage of N/HMPs who agreed that they should integrate with a range of different conventional primary health care practitioners was: 89% with regards to GPs; 87% with regards to specialists; 73% with regards to physiotherapists; 91% with regards to midwives; 82% with regards to nurses; 74% with regards to dietitians; and 81% with regards to clinical psychologists. The average number of referrals per year made by each N/HMP to particular conventional primary health care providers was as follows: 12.0 to GPs (representing 3.3% of annual patient visits) ; 7.1 to specialists (1.9%) ; 7.5 to physiotherapists (2.1%); 7.9 to midwives (2.2%); 4.5 to nurses (1.2%); 7.5 to dietitians (2.1%); and 4.8 to clinical psychologists (1.3%). The average number of referrals per year received by each participant from particular conventional primary health care practitioners was as follows: 10.1 from nurses; 8.7 from midwives; 7.1 from GPs; 5.3 from physiotherapists; 4.7 from clinical psychologists; 3.5 from specialists; and none from dietitians.

The following reasons were given by the N/HMPs as to why they referred to conventional primary health care practitioners: to treat conditions outside their own scope of practice.

(70%); in recognition of early warning signs that require further investigation (77%); for medical diagnosis of a condition (72%); for conditions that are more suited to other treatments beyond their own (63%); for conditions too severe to deal with (62%); for conditions that have not responded to current naturopathic and herbal medicine treatment (37%); to gain clearance or rule out contraindication for N/HM treatment (37%); for conditions not covered by their own training (33%); and for conditions for which N/HMP had limited success (21%). However, N/HMPs referrals to and from conventional providers were primarily by word of mouth with 85% of referrals to conventional practitioners and 75% of referrals from conventional practitioners being via this method.

Tables 3, 4, 5 and 6 report the relationships between referrals from N/HMPs to selected conventional practitioners and: age; years of experience; average case-loads; and academic qualifications. A significant relationship exists between referrals to specialists and average case-loads (of N/HMPs) per month (p = 0.02), as well as between age and referrals to clinical psychologists (p = 0.062).

**Table 3:** **The association between practitioner characteristics and referrals made between naturopathic and herbal medicine practitioners and GPs**

**Practitioner and practice-related characteristics**		**Annual number of referrals made to a GP**			**Annual number of referrals received from a GP**		
		**<6 (n = 35)**	**6 or more (n = 45)**	**p-value**	**<6 (n = 37)**	**6 or more (n = 23)**	**p-value**
		**Mean (SD)**	**Mean (SD)**				
**Years of experience**		12.1 (10.3)	10.1 (8.2)	0.346	2.9 (1.6)	3.3 (1.5)	0.275
**Average case-load per month (all)**		23.8 (22.6)	39.4 (33.8)	0.025	33.9 (24.4)	41.6 (36.0)	0.298
		**%**	**%**		**%**	**%**	
**Age**	**22-44**	37.1	40.9	0.733	25.0	26.1	0.925
	**>44**	62.9	59.1		75.0	73.9	
**Education**							
Diploma		50.0	42.9	0.130	34.5	53.3	0.261
Under-graduate		39.3	25.7		44.8	20.0	
Post-graduate		10.7	31.4		20.7	26.7

**Table 4:** **The association between practitioner characteristics and referrals made from naturopathic and herbal medicine practitioners to specialists**

**Practitioner and practice-related characteristics**		**Annual number of referrals made to a specialist**		
		**<6 (n = 30)**	**6 or more (n = 9)**	**p-value**
		**Mean (SD)**	**Mean (SD)**	
**Years of experience**		11.5 (9.4)	8.5 (7.1)	0.413
**Average case-load per month (all)**		26.6 (19.0)	49.0 (36.1)	0.020
		**%**	**%**	
**Age**	**22-44**	40.0	37.5	0.898
	**>44**	60.0	62.5	
**Education**				
Diploma		45.4	57.1	0.532
Under-graduate		36.4	14.3	
Post-graduate		18.2	28.6

**Table 5:** **The association between practitioner characteristics and referrals made between naturopathic and herbal medicine practitioners and physiotherapists**

**Practitioner and practice-related characteristics**		**Annual number of referrals made to a physiotherapist**			**Annual number of referrals received from a physiotherapist**		
		**<6 (n = 23)**	**6 or more (n = 6)**	**p-value**	**<6 (n = 12)**	**6 or more (n = 6)**	**p-value**
		**Mean (SD)**	**Mean (SD)**				
**Years of experience**		12.0 (10.2)	5.5 (6.8)	0.160	2.8 (1.8)	3.3 (1.4)	0.559
**Average case-load per month (all)**		38.7 (29.4)	24.7 (15.1)	0.275	43.1 (23.0)	31.0 (44.9)	0.455
		**%**	**%**		**%**	**%**	
**Age**	**22-44**	34.8	50.0	0.494	50.0	16.7	0.171
	**>44**	65.2	50.0		50.0	83.3	
**Education**							
Diploma		50.0	25.0	0.450	42.9	33.3	0.961
Under-graduate		43.8	50.0		28.6	33.3	
Post-graduate		6.2	25.0		28.6	33.4

**Table 6:** **The association between practitioner characteristics and referrals made between naturopathic and herbal medicine practitioners and Midwives**

**Practitioner and practice-related characteristics**		**Annual number of referrals made to a midwife**			**Annual number of referrals received from a midwife**		
		**<6 (n = 22)**	**6 or more (n = 16)**	**p-value**	**<6 (n = 20)**	**6 or more (n = 17)**	**p-value**
		**Mean (SD)**	**Mean (SD)**				
**Years of experience**		12.8 (10.2)	9.8 (7.4)	0.346	2.7 (1.9)	2.9 (1.5)	0.685
**Average case-load per month (all)**		35.1 (28.8)	36.3(30.7)	0.909	29.0 (27.1)	40.0 (27.4)	0.234
		**%**	**%**		**%**	**%**	
**Age**	**22-44**	29.2	56.3	0.087	30.0	41.2	0.478
	**>44**	70.8	43.7		70.0	58.8	
**Education**							
Diploma		41.2	50.0	0.114	40.0	45.4	0.497
Under-graduate		41.2	8.3		40.0	18.2	
Post-graduate		17.6	41.7		20.0	36.4	

A majority of the N/HMPs surveyed (60%) did not utilise electronic medical records/client data sheets in their practice while some (31%) reported using both electronic and paper-based clinical records. Only 4% of the respondents employed a purely electronic medical records/client data system. Respondents were asked what information they would find useful if they had access to patients’ medical records. The responses were as follows: prescribed medication (94%); results of diagnostic tests (94%); medical history (90%); surgical information (90%); medical diagnosis (89%); immunisation history (80%).

### Contribution to national health objectives

The N/HMPs were asked to assess whether they perceived their practice as being aligned to the NZ Ministry of Health published public health objectives [16]. The areas where the N/HMPs perceived they made their greatest contributions were in: improving nutrition (93%); increasing physical activity (85%); reducing obesity (82%); reducing smoking (79%); reducing the incidence and the impact of CVD (79%); reducing the incidence and the impact of diabetes (78%); and reducing the incidence and the impact of cancer (68%).

**Figure 1:**
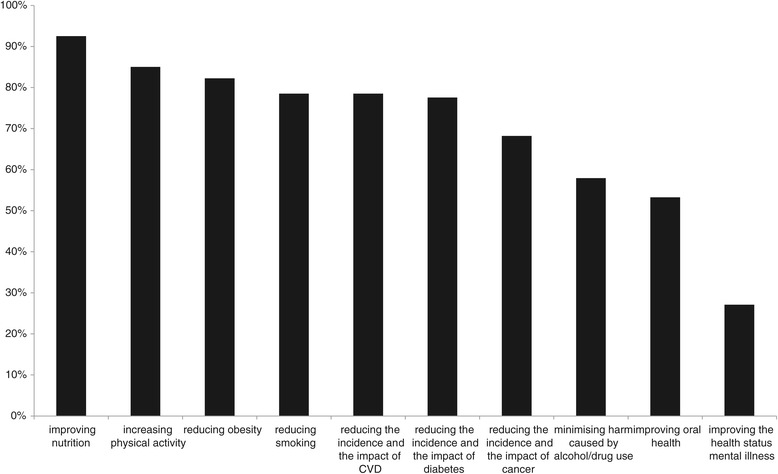
N/HMPs perceived alignment with NZ national health goals.

See Figure 1 N/HMPs perceived alignment with NZ national health goals.

## Discussion

This paper reports the first in-depth examination of N/HMP practice in NZ. The study identified a number of interesting findings.

### Demographics

The results suggest that N/HMPs are predominantly older than 35. This may be a consequence of lack of registration leading to lack of career opportunities, or N/HMP being seen as a ‘secondary’ occupation by older women mainly.

### Integrative practices by N/HMPs

The data in our study suggests that referrals between N/HMPs and conventional care are occurring regularly (albeit on an informal basis). This is similar to referral practices reported between HMPs and conventional care in Australia [18], as well as other countries [19], but there is little data concerning the method of referral internationally or in NZ. The informality of referral mechanisms is highlighted in our survey results. Referral by word of mouth from N/HM to conventional care (and vice versa) is particularly predominant. This is an important area for future research examination, as there is potential, within a purely patient mediated system of referral such as word of mouth, for a lack of information (or misinformation) being communicated by the patient to either practitioner which could compromise safety (particularly in drug-herb or drug-nutrient interactions) and effectiveness (particularly because of the lack of access to diagnosis and treatment information between practitioners). However, the prevalence of referral for conditions outside the N/HMPs scope of practice indicates a degree of self-regulation around safety issues in relation to interaction with conventional care. This finding could be confirmed by a more in-depth study of referral perceptions and practices between N/HMPs and GPs. Whilst several important studies have investigated referrals between conventional care and CAM [13,18-26], the method of referral has seldom been identified and described. A study of Australian and NZ acupuncturists has identified word of mouth as the main referral method [25], which is consistent with the findings in our survey. Another NZ study of osteopaths found that referral was primarily facilitated through patient networks, with word of mouth recommendation highly valued [26]. In many instances the GP will primarily refer at the request of the patient [20,23,27] despite the reluctance of many patients to discuss CAM options with their GP [20,28].

No statistical correlations were found between N/HMPs referrals to GPs or referrals received by N/HMPs from GPs and age, practice years and academic qualifications (Table 3). Those practitioners with a high monthly caseload made more referrals to GPs (p = 0.025) and specialists (p = 0.020), compared to those with a low monthly caseload (Tables 3 and 4). The implications of these results for N/HMPs need further investigation, but they may possibly indicate that those N/HMPs’ who practice full time have a greater understanding of the need to develop integrative networks with a variety of conventional practitioners.

No statistical correlations were found between referral to other conventional practitioners and age, practice years or academic qualifications (Tables 5, 6 and 7). It is unclear as to whether these referral patterns reflect the attitudes to N/HM from conventional practitioners. An increase in referrals could potentially be facilitated by greater recognition, as lack of statutory registration, in Australia, has been shown to be a hurdle to referrals to CAM practitioners [29].

**Table 7:** **The association between practitioner characteristics and referrals made between naturopathic and herbal medicine practitioners and Clinical Psychologists**

**Practitioner and practice-related characteristics**		**Annual number of referrals made to a clinical psychologist**			**Annual number of referrals received from a clinical psychologist**		
		**<6 (n = 26)**	**6 or more (n = 12)**	**p-value**	**<6 (n = 11)**	**6 or more (n = 4)**	**p-value**
		**Mean (SD)**	**Mean (SD)**				
**Years of experience**		14.0 (2.2)	9.3 (7.9)	0.196	3.4 (1.9)	4.3 (0.5)	0.374
**Average case-load per month (all)**		37.0 (26.2)	45.8 (29.1)	0.367	35.5 (21.3)	76.6 (54.8)	0.047
		**%**	**%**		**%**	**%**	
**Age**	**22-44**	23.1	54.6	0.062	27.3	50.0	0.409
	**>44**	76.9	45.4		72.7	50.0	
**Education**							
Diploma		55.6	57.1	0.446	100.0	66.7	0.168
Under-graduate		33.3	14.3		0.0	0.0	
Post-graduate		11.1	28.6		0.0	33.3	

### Registration and regulation

Our study findings showing that practitioners favour registration is not dissimilar from data on N/HMPs’ preferences reported in Australia [10]. Most of the practitioners in our study favoured a mix of government and private medical insurance funding for N/HM. However, more investigation needs to be carried out to determine if funding is a major driver influencing practitioners’ perceptions towards seeking statutory registration. A strong correlation exists between N/HMPs favouring integration and favouring registration implying that, in the perception of N/HMPs, these two issues may be linked. This perception by N/HMPs may be justified by the favourable attitudes and referral patterns of NZ GPs to osteopaths and chiropractors (statutory registered CAM modalities), which indicate a degree of acceptance and integration [11].

Statutory registration of CAM modalities worldwide is supported by the World Health Organisation in its Traditional Medicine Strategy [30] and patient use of N/HMPs’ services would appear to create some impetus for such registration [31].

Internationally there is a move towards registration of N/HMPs in a number of countries [32-35]. The US and Canada have licensing (registration) of naturopaths in a number of states [31]. International harmonisation of standards of education and practice for N/HMPs has the potential to create an environment that supports moves towards registration in NZ. This would have potential for greater employment outcomes for N/HMPs, particularly if their perception of alignment with the NZ Health Strategy [16] goals proves to be valid.

### Research

Our study results indicate that whilst research is valued and perceived by N/HMPs to have an impact on practice, many N/HMPs acknowledged they had no skills in interpreting research. This lack of research literacy could be reflected in the prevalence of reliance on anecdotal (client reported) assessment of treatment effectiveness. It is possible that a general lack of familiarity with research methodology could affect N/HMP’s perspectives, and lead to the reliance on manufacturer’s seminars and trade newsletters as sources of researched information about N/HMP treatments. Internationally, studies have shown that CAM practitioners rely on secondary sources of researched information, such as texts, traditional theory and practice, the internet, manufacturer’s information and information from educators [12,18,36-39]. The reliance on industry information among N/HMPs is not dissimilar to the results of 3^rd^ and 4^th^ year medical residents in the US, This study showed that those who were reliant on pharmaceutical industry sources for their prescribing behaviours were less likely to utilise researched evidence in practice and recommends educational interventions to reverse this situation [40]. It is possible N/HMPs favour approaches to treatment that incorporate clinical experience, traditional information and patient circumstances to guide treatment, rather than relying on research as a primary source of evidence for practice [41-44]. A survey of CAM practitioners (including 68 N/HMPs) attitudes and use of evidence in Australia found that published clinical evidence ranked fourth in information source preference with traditional knowledge, texts and clinical practice guidelines being given priority. Similar to the current study lack of skills was given a main reason for not implementing evidence in practice. Other reasons were lack of evidence sources, time and industry support [44]. It must be noted however that this attitude is not limited to N/HMPs. A systematic review of the barriers to GPs use of evidence-based medicine found that GPs utilised clinical preference and experience, as well as patient’s situations and the practice setting over a purely ‘evidence-based’ approach [45]. An 2008 NZ study of practice nurses showed that there was still a lack of knowledge of evidence-based clinical guidelines, with variable use among those who had that knowledge [46] indicating a similarity between N/HMPs and nurses in their attitude to evidence and evidence-based medicine. N/HMPs attitude to research and evidence-based medicine could also be a result of current educational provision in N/HMP, with a lack of commitment to developing research capacity and outputs [35,47,48]. A national CAM research unit (as favoured by the N/HMPs surveyed) has the potential to address some of current research challenges that appear to face the sector in NZ.

One limitation of our study is the thirty-two per cent response rate. However this is typical for health workforce studies of this kind [39], particularly those conducted on-line [49]. We selected a convenience sample to capture those practitioners who belonged to active organisations. We considered that their views would be indicative of those N/HMPs who could truly represent the practices and perceptions of the majority of the professions. Another possible limitation is that it is unclear as to whether referral data was based on case-records or N/HMPs recollection, as the question simply asked the practitioners to state the numbers.

### Contribution to national health objectives

The results suggest that N/HMPs consider that their practices are effective in contributing significantly towards NZ meeting its National Health Strategy [16]. Some preliminary research from the US suggests that CAM is utilised by a section of the population for disease prevention, with naturopathy used by 0.3% [50] with one study suggesting 25% of the US population utilises CAM for health promotion compared to 17% utilisation for treatment purposes [51]. In NZ, a study of CAM use in paediatric patients found that an average of 7% used CAM for prevention purposes (n = 70) [52]. NM has been shown to have potential in prevention of cardiovascular disease (one of the NZ national health objectives) in a pragmatic randomised controlled trial in Canada [53]. In a US review of the role of CAM under the Affordable Care Act, Thompson and Nichter [54] argue that CAM practitioners are well placed to provide cost-effective “preventive and promotive” health services. This argument is echoed (with specific reference to NM in India) by Tripathy [55], Kraft [32] Whilst these results are significant, research into N/HM in health promotion and prevention of disease is in its infancy. The Naturopathic Medical Research Agenda project [56] prioritises research into NM’s role in “… conditions with the highest burden of illness” and “…existing and emerging public health significance”, suggesting that funding and resources need to be directed towards health promotion and disease prevention. The perception of NZ N/HMs of their contribution towards NZs health goals indicates that funding and resources could be well spent in such research.

## Conclusions

N/HMPs are popular with clients in NZ and they appear to desire a closer and more integrated relationship with the conventional medical profession and public health care system. It is important that practitioners across both conventional and N/HM practice develop an understanding of why their patients are utilising both types of health care. Further research is needed to provide rich in-depth exploration of naturopathic and herbal medicine practitioners and their relationship with patients, conventional providers and the provision of publically-funded health care in NZ.

### Endnotes

^a^The terms ‘diploma’ and ‘advanced diploma’ refer to educational levels below undergraduate degree. Advanced diploma is at a level above diploma. Most diploma qualifications are three to four years.

^b^The term ‘research’ indicates ‘researched information’ unless stated otherwise.

^c^‘Statutory registration’ refers to regulation that is registered by an act of parliament, as compared to ‘voluntary registration’ which is governed by a professional bodies without Government oversight.
